# Construction of Complex Features for Computational Predicting ncRNA-Protein Interaction

**DOI:** 10.3389/fgene.2019.00018

**Published:** 2019-02-01

**Authors:** Qiguo Dai, Maozu Guo, Xiaodong Duan, Zhixia Teng, Yueyue Fu

**Affiliations:** ^1^School of Computer Science and Engineering, Dalian Minzu University, Dalian, China; ^2^Dalian Key Laboratory of Digital Technology for National Culture, Dalian Minzu University, Dalian, China; ^3^School of Electrical and Information Engineering, Beijing University of Civil Engineering and Architecture, Beijing, China; ^4^School of Information and Computer Engineering, Northeast Forestry University, Harbin, China; ^5^Department of Hematology, The First Affiliated Hospital of Harbin Medical University, Harbin, China

**Keywords:** ncRNA-protein interaction, complex feature, feature construction, feature selection, random forest

## Abstract

Non-coding RNA (ncRNA) plays important roles in many critical regulation processes. Many ncRNAs perform their regulatory functions by the form of RNA-protein complexes. Therefore, identifying the interaction between ncRNA and protein is fundamental to understand functions of ncRNA. Under pressures from expensive cost of experimental techniques, developing an accuracy computational predictive model has become an indispensable way to identify ncRNA-protein interaction. A powerful predicting model of ncRNA-protein interaction needs a good feature set of characterizing the interaction. In this paper, a novel method is put forward to generate complex features for characterizing ncRNA-protein interaction (named CFRP). To obtain a comprehensive description of ncRNA-protein interaction, complex features are generated by non-linear transformations from the traditional k-mer features of ncRNA and protein sequences. To further reduce the dimensions of complex features, a group of discriminative features are selected by random forest. To validate the performances of the proposed method, a series of experiments are carried on several widely-used public datasets. Compared with the traditional k-mer features, the CFRP complex features can boost the performances of ncRNA-protein interaction prediction model. Meanwhile, the CFRP-based prediction model is compared with several state-of-the-art methods, and the results show that the proposed method achieves better performances than the others in term of the evaluation metrics. In conclusion, the complex features generated by CFRP are beneficial for building a powerful predicting model of ncRNA-protein interaction.

## 1. Introduction

The DNA component encyclopedia project (ENCODE) has revealed that most of RNAs in the human transcriptome are non-coding RNAs (ncRNA), which are not involved in coding protein (ENCODE Project Consortium, 2012). As a kind of critical regulatory molecules, ncRNA can regulate gene expression in different stages, such as epigenetic inheritance, transcription and post-transcription (Quan et al., 2015; Zeng et al., 2017). It participates in various cellular processes such as chromatin modification, transcriptional regulation, translation and post-translational modification (Yarmishyn and Kurochkin, 2015; Yotsukura et al., 2016). Increasing evidences show that ncRNA is closely related to many major diseases that seriously endanger human health and life (Chen et al., 2013; Tang et al., 2017). However, the functional mechanisms of most ncRNAs remain to be further studied and determined. It is worth noting that many ncRNAs often perform their functions by forming RNA-protein complexes (Zhu et al., 2013). For examples, as a scaffolding molecule, HOTAIR RNA combines with PRC2 and LSD1 protein complexes at its 5′ and 3′ ends, respectively, to involve in histone methylation (Tsai et al., 2010). It has been found that over-expression of HOTAIR could induce the relocation of PRC2 protein complex in the whole genome, which could silence tumor suppressor genes and thus promote the development and metastasis of malignant tumors such as breast cancer and liver cancer (Gupta et al., 2010). Xist RNA, which regulates X chromosome inactivation, can interact with more than 80 proteins in many biological processes (Chu et al., 2015). It can be found that there are pervasive synergistic relationships between ncRNAs and proteins, which play important roles in cellular activities and disease regulations. Therefore, determining the interactions between large amount of ncRNAs and proteins is of great significance for revealing molecular mechanisms of ncRNAs in human diseases and biological processes.

Recently, experiment techniques such as CLIP-sep, RIP-seq and fRIP-seq have been developed for uncovering ncRNA-protein interactions (Ferrè et al., 2016). Many significant findings have been obtained by using these methods. However, it still remains some challenges such as expensive, time-consuming and labor-intensive (Luo et al., 2017). Therefore, it is important to develop an accurately computational method for predicting ncRNA-protein interactions to provide valuable supports and supplements for revealing functionalities of ncRNAs. Computational prediction of ncRNA-protein relationship has attracted much attention in the fields of ncRNA and computational biology. It can be roughly divided into the prediction of the interaction pairs and the prediction of binding sites. The former refers to the method of predicting the interacting relationship between ncRNA and protein. The latter approach focuses on the interaction between amino residues in protein and nucleic acid bases in RNA. In this paper, we focus on the prediction methods of interaction between ncRNA and protein molecules. Previous methods for predicting ncRNA-protein interaction could be roughly divided into machine learning based method and network based method. The machine learning based predicts novel ncRNA-protein interactions by training a machine learning model on available known interaction data (Ferrè et al., 2016). The network based usually constructs a heterogeneous network with known ncRNA-protein interactions and predicts novel edges between ncRNA and protein nodes within the network by using some link prediction algorithm as in Zhang et al. (2018). In this study, we focus on the machine learning based method, because it is able to predict the interaction between molecules that are not present in the training data.

**Figure 1:**
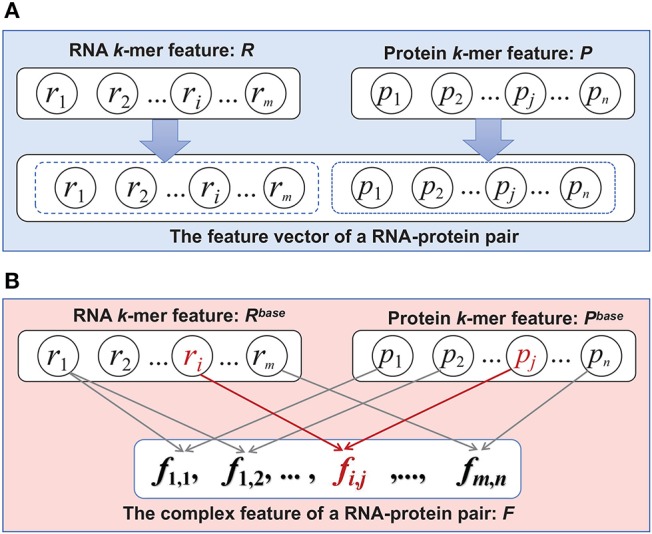
The schematic diagram of the traditional feature and the proposed complex feature. **(A)** Traditional feature extraction. **(B)** Complex features construction.

Many machine learning based methods have been developed for predicting ncRNA-protein interaction (Lu et al., 2013; Yang et al., 2013) in the past decade. For example, catRAPID proposed in Bellucci et al. (2011) is one of the earliest machine learning based methods for predicting ncRNA-protein interactions. It extracts sequential features from the primary sequences of a ncRNA-protein pair and trains prediction model based on support vector machine and random forest. Cheng et al. (2015) proposed a method named PRIPU, which built a biased support vector machine model to tackle the imbalance problem of positive and negative samples in the dataset. Pan et al. (2016) employs deep learning model with stacked ensemble technique. Most of previous methods focused on using more powerful machine learning algorithms. In fact, in addition to machine learning model, how to extract a set of good features that could appropriately characteristic the properties of samples is another critical problem for improving the predicting performance (Zou et al., 2016b). It is worth noting that developing a powerful feature extraction method for a specific set of samples usually needs to consider what field the sample comes from. It is because that in different fields the properties of actual objects corresponding to the samples may be very different. In other words, the properties of the samples itself should be particularly considered when generating features for training a prediction model. With respect to the interaction between ncRNA and protein, each sample in the dataset is composed of two primary sequences that corresponds to a pair of ncRNA and protein molecules, respectively. When characteristic ncRNA-protein pair, most of existing methods typically extract the sequential feature vectors from the two molecules separately, and then directly concat the two feature vectors together into one vector that is finally taken as the feature of the given pair. For example, as in Figure 1A, for a pair of ncRNA and protein, their *k*-mer feature vectors are first extracted, which we denoted as *R* and *P*, respectively. Then, *R* and *P* are directly concatenated to the feature vector of the ncRNA-protein pair. Obviously, it is an easy way to characteristic the pair that is composed of two distinct molecules. However, it is worth noting that this kind of simple feature concatenation does not consider the correlation between the two molecular features, which may be critical for understanding the interactive properties of these molecules. In fact, the interaction between a pair of RNA and protein is usually formed by the physical contacts between the amino acid residues and nucleotide bases at the interface (Hudson and Ortlund, 2014). Consequently, to characterize the interaction between the two molecules, it should not only have to extract the separate features from individual molecules, but also need to focus on the complex relations between these features.

In this paper, we propose a framework for constructing Complex Features to predict ncRNA-Protein interactions (CFRP). The complex features are generated by employing some fusion methods upon the traditional individual molecule features (base features) of ncRNA and protein. The motivation behind constructing the complex feature is to emphasize the complex relations between the basic features of different molecules, and thus to characterize the interactive activities between a pair of RNA and protein. In particular, complex features are constructed by using one or more non-linear operations on the two base features. As in Figure 1B, let *r*_*i*_ be an element in RNA base feature and *p*_*j*_ in protein base feature. A complex feature *f*_*i,j*_ is the result of conducting a specific non-linear operation on *r*_*i*_ and *p*_*j*_. A variety of different non-linearities were investigated for constructing the complex feature in this work, in an attempt to comprehensively characterize the interactive properties between the two molecules. Furthermore, a feature selection based random forest (RF) are employed to reduce the dimensions of constructed complex feature, which make it concise and efficient for training predicting models. To investigate the effectiveness of the proposed CFRP method, the complex features constructed by the method are employed to train a machine learning model (CFRP model) for predicting ncRNA-protein interactions. We conducted extensive tests against to CFRP model on several widely used public datasets. The experimental results demonstrated that the complex feature constructed by CFRP method is helpful to obtain a good prediction model with better performance than the traditional k-mer feature. Compared with other state-of-the-art methods, CFRP model can achieve better prediction performance in terms of many metrics. Especially, on the *Sum* metric, CFRP method is superior to other methods on all data sets. In conclusion, CFRP can produce a set of discriminative features against to the task of predicting ncRNA-protein interactions.

## 2. Methods

**Figure 2:**
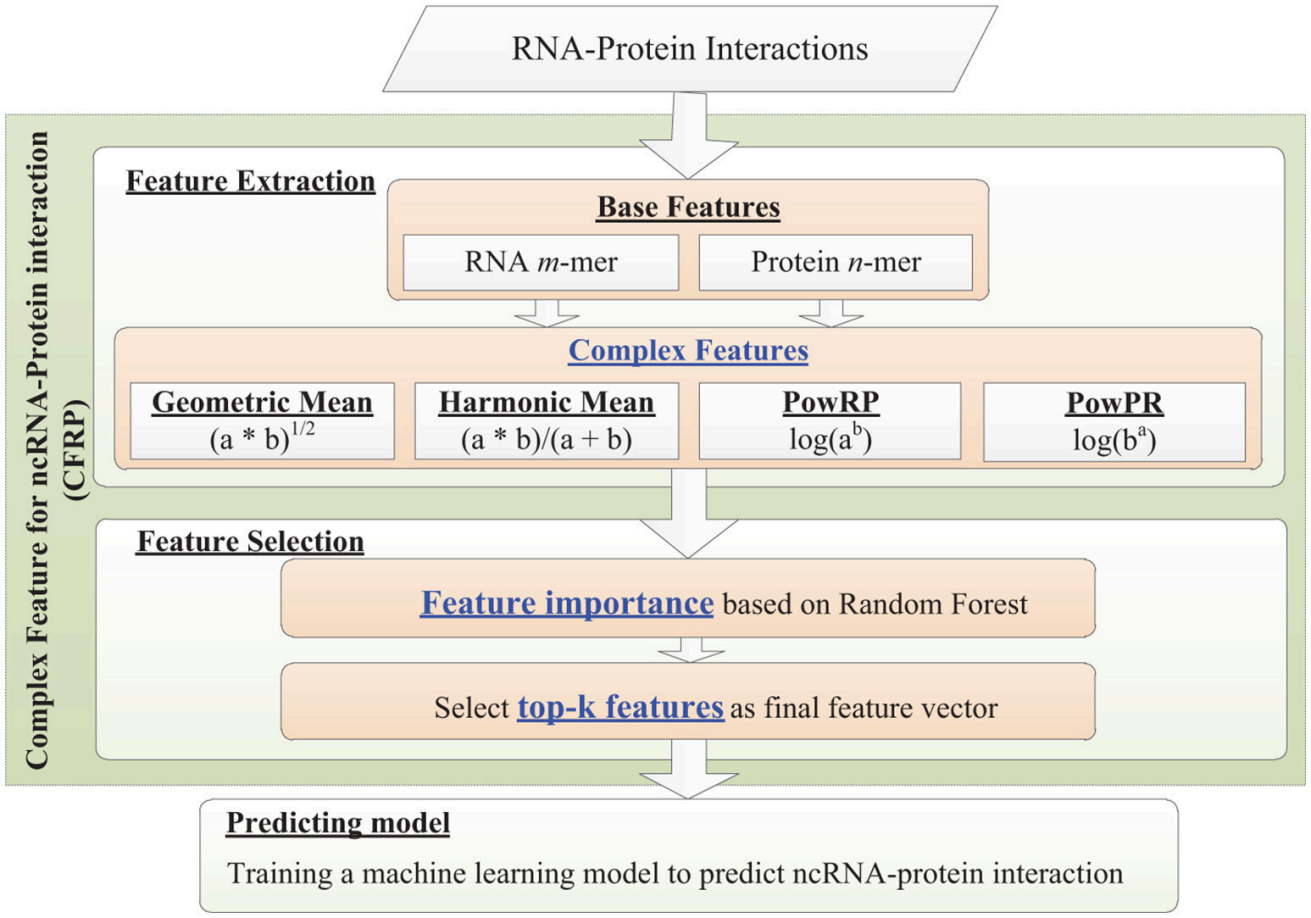
The flowchart of constructing interactive complex features.

In this section, a novel framework for constructing Complex Feature that is used to characterize ncRNA-Protein interaction (CFRP) is put forward, which generates complex feature by employing a set of non-linear transformations upon the traditional k-mer sequential feature (base feature). As shown in Figure 2, the framework consists of several steps including base feature extraction, complex feature generation, feature ranking and selection. Specifically, CFRP firstly extracts traditional k-mer features from a pair of RNA and protein as their base features, respectively; then, a set of complex features are constructed by employing different kinds of non-linear transformations upon the extracted base features; finally, the generated complex features are ranked by the feature importances that are induced from a trained random forest model and then the top-k important features of them are chosen as the final feature of input ncRNA-protein pair. The generated complex feature could be used to train a powerful prediction model for ncRNA-protein interaction.

### 2.1. Base Feature Extraction

Given the sequences of a ncRNA-protein pair, we first extract k-mer features from them, respectively, as their base features. For the RNA, *m*-mer feature is extracted from its primary sequence, where *m*-mer represents the frequency of each kind of successive *m* base combination in the sequence. As there are four kinds of nucleotides (A, C, G, U) in RNA, a base feature vector *R*^*base*^ (Equation 1) has 4^*m*^ dimensions.

(1) $$\begin{array}{l} R^{base}=\left\{r_{1},r_{2},\ldots ,r_{i},\ldots ,r_{4^{m}}\right\} \end{array}$$

For the protein, there are 20 kinds of amino acid residues existing in its primary sequence. Let *n* be the length of k-mer for protein. If directly computing *n*-mer frequency, we will get a feature vector with 20^*n*^ dimensions. Such a number of dimensions is too expensive for subsequent construction of complex feature. To reduce the dimensions of protein base feature vector, we group 20 kinds of residues into several subsets as Shen et al. (2007). They proposed a 7-group strategy to classify 20 amino residues into 7 groups based on their physiochemical properties, as {A, G, V}, {I, L, F, P}, {Y, M, T, S}, {H, N, Q, W}, {R, K}, {D, E}, and {C}. This grouping method could reduce the computational cost without significantly performance reduce on characterizing protein sequence. By this grouping, the original protein sequence could be translated into a new string that is composed of 7 characters. Then, a 7^*n*^ dimensional *n*-mer vector *P*^*base*^ could be extracted (Equation 2) from the new string as the base feature of input protein. Each element *p*_*j*_ in the vector is the frequency of a certain *n*-mer in the translated string.

(2) $$\begin{array}{l} P^{base}=\left\{p_{1},p_{2},\ldots ,p_{j},\ldots ,p_{7^{n}}\right\} \end{array}$$

The base feature vectors extracted from RNA and protein (*R*^*base*^ and *P*^*base*^) are normalized by the sequence lengths of RNA and protein, respectively.

### 2.2. Complex Feature Construction

To represent the complex relation between ncRNA and protein, a set of complex features is generated by introducing non-linear transformations upon the base features of individual RNA and protein sequences. In particular, some non-linear operations such as geometric mean, harmonic mean and power operation are introduced. As shown in Figure 2, given base features *R*^*base*^ and *P*^*base*^ of a pair of RNA and protein, a set of complex features such as *GM, HM, PowRP*, and *PowPR* is generated. The details are as follows:

the *GM* feature,

(3) $$\begin{array}{l} GM=\left\{gm_{ij}|1\leq i\leq 4^{m},1\leq j\leq 7^{n}\right\} \end{array}$$

where *gm*_*ij*_ is the geometric mean of *r*_*i*_ and *p*_*j*_ as

$$gm_{ij}=\sqrt{r_{i}\times p_{j}},$$

the *HM* feature,

(4) $$\begin{array}{l} HM=\left\{hm_{ij}|1\leq i\leq 4^{m},1\leq j\leq 7^{n}\right\} \end{array}$$

where *hm*_*ij*_ is the harmonic mean of *r*_*i*_ and *p*_*j*_ as

$$hm_{ij}=2\left(r_{i}\times p_{j}\right)/\left(r_{i}+p_{j}\right),$$

and power operation features,

(5) $$\begin{array}{l} PowRP=\left\{pr_{ij}=\log r_{i}^{p_{j}}|1\leq i\leq 4^{m},1\leq j\leq 7^{n}\right\} \end{array}$$

(6) $$\begin{array}{l} PowPR=\left\{pp_{ij}=\log p_{j}^{r_{i}}|1\leq i\leq 4^{m},1\leq j\leq 7^{n}\right\} \end{array}$$

By means of above different non-linear operations, we could get a 4 × 4^*m*^×7^*n*^-dimensional feature vector that consists four kinds of complex features. These raw CFRP features could be used to describe the interactive activities between RNA and protein in a more comprehensive view.

### 2.3. Feature Selection

Complex features with thousands of dimensions might arise the problem of dimensionality curse. The high dimensional feature space will yield several problems such as data sparseness, over-fitting of prediction model and high computational cost (Li et al., 2016). In order to reduce the adverse effect, a feature selection method should be conducted on the high dimensional space to select the features with more information values and to remove those ones with less importances. It has been proven to be effective and efficient for solving variety machine learning problems on high-dimensional data (Zou et al., 2016a). In this work, the strategy of feature selection against to the complex features constructed above is a two-step process: the first step is to rank all features in descending order with respect to the importance according to their contribution to the classification, and then the top-k important features is selected as the final features. To get the importance of feature, we employed a random forest model (RF) based model. As known, in a decision tree, features used at the top of a decision tree are considered to be contribute to a larger fraction of the input samples for the prediction task. Consequently, we could estimate the importance of the feature based on its contribution to the prediction in the tree. Random forest are composed of a set of decision trees. By estimating the average value of feature importance on multiple trees in a forest, we could get the importance of features with lower variance for a given prediction task (Zhou et al., 2016). After the estimation of feature importance, we could get top-k features according the importance as the final features used for characterizing RNA-protein interactions.

### 2.4. Training a CFRP Model

The CFRP complex features could be used to train a predicting model for ncRNA-protein interaction on a certain dataset. In general, a common procedure for training a CFRP-based predicting model is as:

- Extracting *m*-mer and *n*-mer features from RNA and protein primary sequences, respectively, as their base features;
- Generating complex features based on the base features as section 2.2;
- Selecting the top-k complex features with respect to the feature importances yielded by a trained random forest model;
- Training a machine learning model with the selected complex features on a dataset.

In accordance with this framework, we trained a CFRP-based model using random forest for the follow experimental testing and performance evaluation.

## 3. Experimental Results

In order to validate the proposed method of constructing complex feature for ncRNA-protein interaction, CFRP model was tested on a set of high-quality public datasets. Several metrics widely used in the field were employed to measure the performance of a prediction model. A series of experimental tests were carried out: (1) the properties of CFRP feature using different k-mer length; (2) the effectiveness of the proposed CFRP feature; (3) comparison of CFRP model with other state-of-the-art methods. CFRP source code and other related resources can be download on the website (http://www.dailab.cn/CFRP/index.html).

### 3.1. Datasets

Three datasets were adopted to test the CFRP method proposed in the experiments, including RPI369 (Muppirala et al., 2011), RPI488 (Pan et al., 2016) and RPI2241 (Muppirala et al., 2011), all of which are widely used in the field of predicting ncRNA-protein interaction (Muppirala et al., 2011; Lu et al., 2013; Pan et al., 2016). All of these datasets consist of non-redundant experimentally-validated ncRNA-protein interaction pairs that are extracted from the three-dimensional structures of RNA-protein complexes within the Protein Data Bank (PDB) (Westbrook et al., 2002). The summary information of these datasets is listed in Table 1. In detail, RPI369 consists of 369 experimentally-validated RNA-protein interactions as positive samples and the same number of negative RNA-protein interactions, in which the negative samples are generated with the randomly pairs of proteins and RNAs that does not present in positive sample set. RPI488 is obtained by Pan et al. (2016), which is extracted from 18 ncRNA-protein complexes also in PDB. It consists of 488 samples including 243 RNA-protein interactions and 245 non-interactions. RPI2241 includes 2,241 experimental-validated RNA-protein interactions and negative ones, respectively, where the method for generating negative samples is same as RPI369.

**Table 1:** ncRNA-protein datasets used in this study.

**Dataset**	**# of interaction pairs**	**# of RNAs**	**# of proteins**	**References**
RPI369	369	332	338	Muppirala et al., 2011
RPI488	488	25	247	Pan et al., 2016
RPI2241	2241	842	2043	Muppirala et al., 2011

### 3.2. Performance Metrics

Several metrics are employed for measuring the performance on predicting ncRNA-protein interaction, including *Accuracy* (*Acc*), *Sensitivity* (*Sen*), *Specificity* (*Spe*), *Precision* (*Pre*), Matthews correlation coefficient (*Mcc*) and area under curve (*AUC*) of the receiver operation Characteristic (ROC). The details of these metrics are as follows,

(7) $$\begin{array}{l} Acc=\frac{TP+TN}{TP+TN+FP+FN} \end{array}$$

(8) $$\begin{array}{l} Sensitivity=\frac{TP}{TP+FN} \end{array}$$

(9) $$\begin{array}{l} Specificity=\frac{TN}{TN+FP} \end{array}$$

(10) $$\begin{array}{l} Precision=\frac{TP}{TP+FP} \end{array}$$

(11) $$\begin{array}{l} Mcc=\frac{TP\times TN-FP\times FN}{\sqrt{\left(TP+FP\right)\left(TP+FN\right)\left(TN+FP\right)\left(TN+FN\right)}} \end{array}$$

where *TP, TN* represent the number of correctly predicted positive and negative samples, respectively, and *FP, FN* represent the number of samples wrongly predicted as positive and negative, respectively. These metrics are widely used for measuring the performance of a predicting model in the field (Bellucci et al., 2011; Cheng et al., 2015; Pan et al., 2016) and other related fields (Su et al., 2018; Wei et al., 2018) in Bioinformatics. In addition, since none of the above metrics is a gold standard, we also investigate the sum of above 6 metrics to measure the overall performance of the prediction model (*Sum*). In order to reduce the variance of performance, all experimental results about CFRP model are obtained by means of k-fold cross-validation (Arlot and Celisse, 2010). In detail, the sample set is divided into *k* sub-sets with equal size. For each model training, one sub-set is taken as the testing sample set and the rest ones as the training set. The average performance metrics of these *k* models are taken as the final result of performance evaluation of the model.

### 3.3. CFRP Feature With Different Length of K-mer

As described, CFRP generates complex feature on the basis of base feature of RNA and protein. That is to say that using different length of k-mer in the base feature will definitely affect the performance and computing efficiency of CFRP method. Therefore, we study some properties of CFRP feature, such as effects on computing cost and discriminative performance, when using different k-mer lengths in base feature.

#### 3.3.1. Time and Memory Consumptions for Constructing CFRP

**Figure 4:**
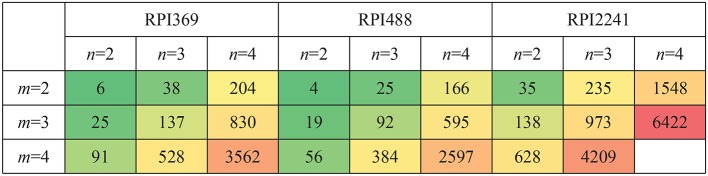
Time consumption (Seconds) of CFRP-models with different k-mer length on three datasets.

**Figure 5:**
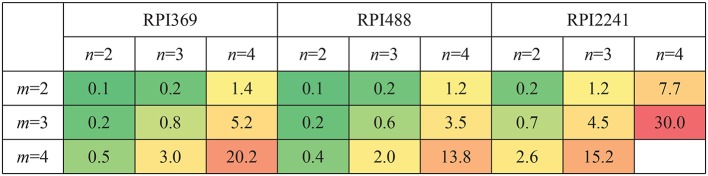
Memory consumption (G) of CFRP-models with different k-mer length on three datasets.

Let *m, n* be the length of k-mer in RNA and protein, respectively. *m* = 2, 3, 4 and *n* = 2, 3, 4 were tested. Complex feature was constructed by CFRP for each setting of *m, n*. The computer used for conducting the experiments was equipped with an E7-4809 v4 CPU, 64G memory, and Ubuntu 16.04 system. Python 3.6.7 and scikit-learn 0.19.1 (Pedregosa et al., 2013) were adopted for algorithm implement. The running time and memory space occupied by CFRP for building complex features on different data sets under different values of *m* and *n* were shown in Figures 4, 5, respectively. As can be seen from the tables, running time and memory size were significantly positively correlated with the values of *m* and *n*. For example, on RPI369 dataset, when *m* = 2 and *n* = 2, only about 6 seconds and 0.1G of memory were consumed; when *m, n* = 4, it consumed about 3,562 s of running time and 20.2 grams of memory. Due to the limited memory size of our computer, we were not able to successfully obtain CFRP feature and train a model on RPI2241 dataset.

#### 3.3.2. Comparison of CFRP-Model Using Different Classifiers

Several classic machine learning techniques such as random forest (RF), support vector machine (SVM) and logistics regression (LR) were employed for training CFRP models. The models were denoted as CFRP-RF, CFRP-SVM and CFRP-LR depending on which learning technique it used. These models were trained on RPI369, RPI488, and RPI2241 datasets under different values of *m, n*. And 3-fold, 10-fold cross-validations were both used for evaluating the performance of models, and the results were shown in Tables 2, 3, respectively. Each value in the tables represents the *Sum* performance value obtained by a model under specific *m* and *n* values on a certain dataset, and the value in bold denotes the best one yielded on each dataset. As shown in the tables, CFRP-RF can achieve better prediction performance than the other two models, suggesting that random forest is an appropriate machine learning technique for the task of predicting ncRNA-protein interaction. It also can be found that the best performance on each dataset is mostly obtained when *m* = 4, except for 3-fold on RPI369 dataset. Therefore, we can infer that the 4-mer RNA sequence pattern is more conducive than that of 2-mer to the characterization of the interactive properties of ncRNA against to protein. Considering that most of the relevant works adopted 10-fold cross-validation, the CFRP-RF running results with the best Sum performance on each dataset in the above 10-fold test were used for subsequent experiments, named CFRP model.

**Table 2:** The effects of different k-mer lengths on the prediction performance (3-fold cross-validation).

**Dataset**	**Methods**	****m**** **= 2**			****m**** **= 3**			****m**** **= 4**		
		**n = 2**	**n = 3**	**n = 4**	**n = 2**	**n = 3**	**n = 4**	**n = 2**	**n = 3**	**n = 4**
RPI369	CFRP-RF	4.121	4.046	4.001	4.075	4.142	**4.169**	4.029	4.158	4.035
	CFRP-SVM	3.960	3.917	3.839	3.873	3.916	3.835	3.965	3.804	3.552
	CFRP-LR	3.988	3.845	3.932	3.765	3.805	3.824	3.745	3.732	3.395
RPI488	CFRP-RF	5.249	5.164	5.179	5.311	5.336	5.234	**5.367**	5.297	5.259
	CFRP-SVM	5.079	5.123	5.142	5.290	5.067	5.285	5.293	5.266	5.262
	CFRP-LR	5.062	4.927	4.932	5.118	5.009	5.148	5.122	4.909	5.021
RPI2241	CFRP-RF	3.556	3.518	3.561	3.706	3.682	3.653	3.753	**3.759**	–
	CFRP-SVM	3.158	3.155	3.043	3.513	3.447	3.449	3.630	3.612	–
	CFRP-LR	3.143	3.137	3.017	3.331	3.273	3.397	3.461	3.456	–

*The values in bold represent the best values obtained by the three methods with different k-mer lengths on a certain dataset*.

**Table 3:** The effects of different k-mer lengths on the prediction performance (10-fold cross-validation).

**Dataset**	**Methods**	****m**** **= 2**			****m**** **= 3**			****m**** **= 4**		
		**n = 2**	**n = 3**	**n = 4**	**n = 2**	**n = 3**	**n = 4**	**n = 2**	**n = 3**	**n = 4**
RPI369	CFRP-RF	4.066	4.102	4.070	4.092	4.094	4.112	4.165	4.075	**4.173**
	CFRP-SVM	3.983	3.773	3.764	3.869	3.702	3.729	3.994	3.756	3.746
	CFRP-LR	3.938	3.762	3.741	3.844	3.627	3.701	3.942	3.640	3.666
RPI488	CFRP-RF	5.209	5.157	5.170	5.250	5.228	5.209	5.214	**5.280**	5.201
	CFRP-SVM	5.107	5.139	5.157	5.166	5.191	5.179	5.192	5.181	5.189
	CFRP-LR	4.816	4.962	5.003	5.121	5.003	5.080	5.148	5.051	4.995
RPI2241	CFRP-RF	3.612	3.575	3.598	3.728	3.687	3.767	**3.846**	3.725	–
	CFRP-SVM	3.408	3.260	3.195	3.493	3.418	3.455	3.692	3.414	–
	CFRP-LR	3.373	3.165	3.163	3.376	3.296	3.382	3.476	3.291	–

*The values in bold represent the best values obtained by the three methods with different k-mer lengths on a certain dataset*.

### 3.4. Study on the Effectiveness of CFRP Feature

**Figure 3:**
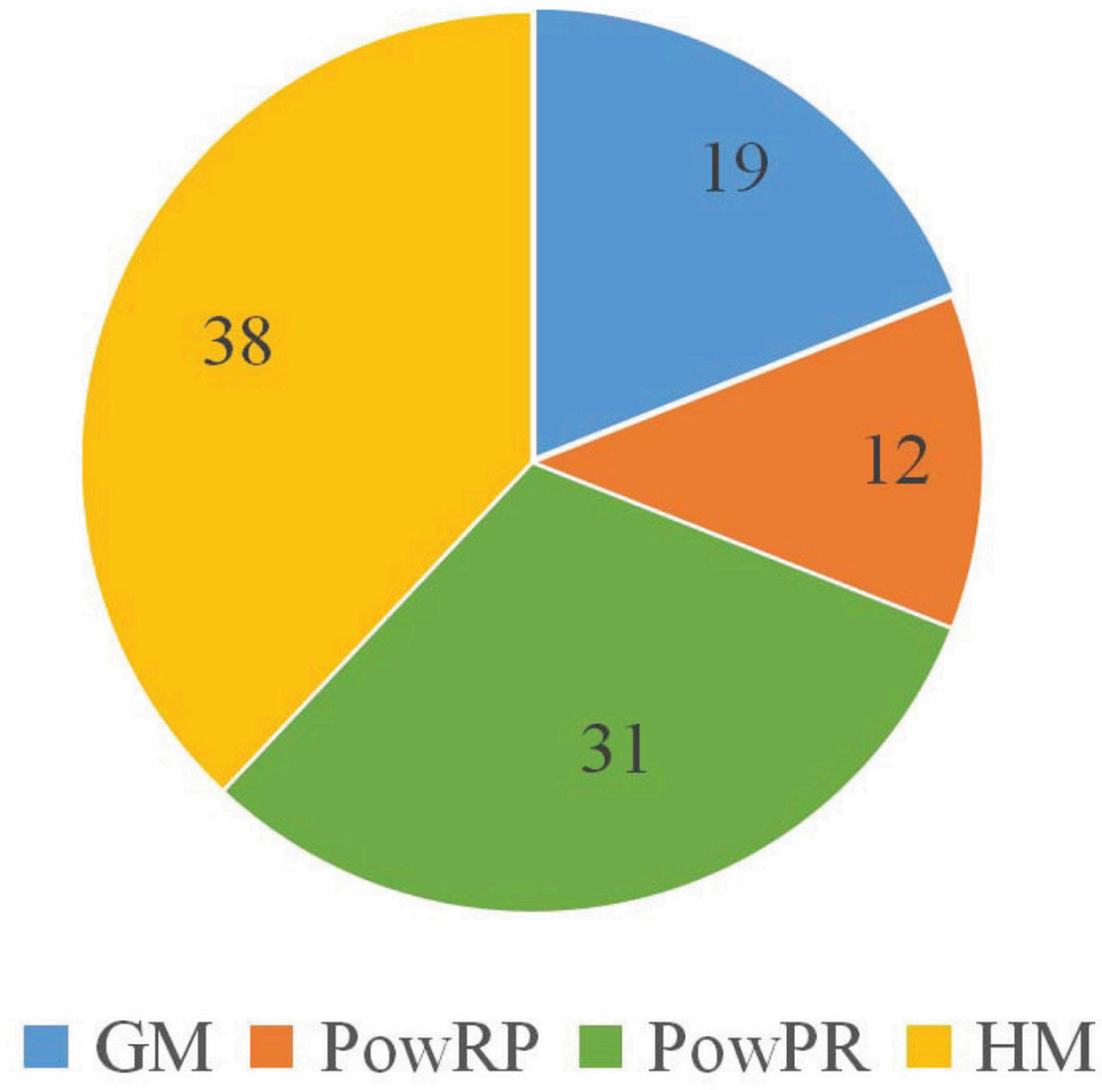
Analysis of the distribution of complex features generated from RPI2241 dataset.

In CFRP, there are four kinds of non-linear transformations that are used to produce GM, PowRP, PowPR and HM complex features. In order to study the effectiveness of different complex features, we analyze the top 100 features generated by CFRP on RPI2241, which are composed of 19 *GM*, 38 *HM*, 12 *PowRP*, and 31 *PowPR* features in Figure 3. It means that the four non-linear operations introduced for generating complex features are all effective, and *HM* is more discriminative for the accurate prediction of ncRNA-protein interactions than other kinds of features.

Furthermore, in order to validate the discriminative ability of CFRP feature, we further trained a model that just uses traditional k-mer feature (base feature) and a model using complex features generated by CFRP but without using any feature selection. These models are also trained with random forest model and denoted as CFRP-raw and BaseFeat, respectively. To investigate different features, we tested CFRP, CFRP-raw and BaseFeat models on RPI369, RPI488, and RPI2241 datasets, the experimental results are shown in Table 4. In the table, the value in bold denotes the best one in the tested models on a certain dataset. As shown, the performance of CFRP-raw on *Sum* is weaker than CFRP model on three datasets and better than the traditional k-mer feature on RPI2241. It suggests that the ability of generated raw complex features for describing ncRNA-protein interaction could be boosted with an appropriate feature selection. Moreover, CFRP model is superior to other two tested models on three datasets for all of performance metrics. For example, the *AUC* indicators of CFRP model on RPI369, RPI488, and RPI2241 are 0.788, 0.938, and 0.744, exceeding 0.028, 0.024, and 0.043 than Basefeat, respectively. In terms of *Accuracy*, CFRP also obtained 0.024, 0.016, and 0.027 improvements over BaseFeat on three datasets. The significant improvements of CFRP-model demonstrate that the proposed CFRP complex feature has a more powerful ability for describing ncRNA-protein interaction than the base feature.

**Table 4:** Performance evaluation of CFRP-model on RPI369, RPI488, and RPI2241.

**Dataset**	**Methods**	**Accuracy**	**Sensitivity**	**Specificity**	**Precision**	**MCC**	**AUC**	**Sum**
	BaseFeat	0.737	0.692	0.708	0.703	0.401	0.760	4.001
RPI369	CFRP-raw	0.728	0.700	0.682	0.688	0.382	0.758	3.937
	CFRP	**0.761**	**0.743**	**0.710**	**0.719**	**0.452**	**0.788**	**4.173**
	BaseFeat	0.926	0.800	0.915	0.905	0.720	0.914	5.180
RPI488	CFRP-raw	0.924	0.794	0.912	0.901	0.712	0.900	5.143
	CFRP	**0.942**	**0.813**	**0.927**	**0.917**	**0.743**	**0.938**	**5.280**
	BaseFeat	0.657	0.618	0.684	0.661	0.302	0.701	3.623
RPI2241	CFRP-raw	0.659	0.597	0.709	0.672	0.308	0.725	3.670
	CFRP	**0.684**	**0.621**	**0.736**	**0.702**	**0.359**	**0.744**	**3.846**

*The values in bold represent the best values obtained by the three methods on a certain dataset*.

### 3.5. Comparison of CFRP Model With Other Methods

In this section, CFRP model is compared with other state-of-the-art methods, including RPISeq (Muppirala et al., 2011) and lncPro (Lu et al., 2013). Similar to CFRP model, these two methods also take primary sequences of RNA and protein as inputs. RPISeq adopts the framework combining traditional k-mer features and random forest, while lncPro predicts ncRNA-protein interaction through scoring the pair by encoding sequences into numeric vectors. The performance of CFRP model is compared with those of RPISeq and lncPro on RPI369, RPI488, and RPI2241 datasets. As illustrated in Table 5, CFRP model performs better than RPISeq and lncPro on three datasets with respect to most of the tested metrics, especially in terms of *Sum* and *AUC* metrics. CFRP model achieves more than 0.179, 0.055, and 0.188 *Sum* improvements than other two methods on three datasets, respectively. In addition, it performs better than other two methods on RPI369 for all of the tested metrics. Also, CFRP gets 0.761, 0.942, and 0.684 *Accuracy* on three tested datasets, which exceeds 0.057, 0.062, and 0.030 at least than other methods, respectively. As a whole, although it is inferior to the other methods on a few indicators, CFRP method performs better than the other two methods in general. It suggests that the method for generating complex features presented in this work is an effective and efficient way to predict ncRNA and protein interaction.

**Table 5:** Comparison between CFRP and other methods on RPI369, RPI488, and RPI2241.

**Dataset**	**Method**	**Accuracy**	**Sensitivity**	**Specificity**	**Precision**	**MCC**	**AUC**	**Sum**
	RPISeq	0.704	0.705	0.702	0.707	0.409	0.767	3.994
RPI369	lncPro	0.704	0.708	0.696	0.713	0.409	0.740	3.970
	CFRP	**0.761**	**0.743**	**0.710**	**0.719**	**0.452**	**0.788**	**4.173**
	RPISeq	0.880	**0.926**	0.822	**0.932**	**0.762**	0.903	5.225
RPI488	lncPro	0.870	0.900	0.827	0.910	0.740	0.901	5.148
	CFRP	**0.942**	0.813	**0.927**	0.917	0.743	**0.938**	**5.280**
	RPISeq	0.646	0.652	0.630	0.663	0.293	0.690	3.574
RPI2241	lncPro	0.654	**0.659**	0.640	0.669	0.310	**0.722**	3.654
	CFRP	**0.684**	0.621	**0.736**	**0.702**	**0.359**	**0.744**	**3.846**

*The values in bold represent the best values obtained by the three methods on a certain dataset*.

To sum up, the above series of experiments show that the CFRP method proposed in this work is effective, and it could produce complex features with better descriptive ability for ncRNA-protein interaction. The main reasons include two aspects. On the one hand, CFRP introduces a variety of complex relations about k-mer base feature, which can characterize the properties of ncRNA and protein interaction from a more comprehensive and higher level. On the other hand, by introducing the feature selection method based on random forest, the dimension of generated complex feature can be significantly reduced, so that the problem of dimensional disaster is avoided. And thus the CFRP feature can be more concise and efficient for training a powerful predicting model.

## 4. Conclusion

The interaction between ncRNA and protein is significant for many critical biological processes and diseases. Developing a powerful computational method for predicting the interaction could provide a important assistance for understanding the molecular mechanism within variety biological activities. When building a prediction model, it is very important to employ a set of features that could effectively characterize the interaction between ncRNA and protein. In this work, we presented a novel framework named CFRP for constructing a set of complex features, which tries to comprehensively characterize interactive activities of a ncRNA-protein interaction. Firstly, k-mer features (base features) are extracted from primary sequences of ncRNA and protein, respectively; secondly, a set of complex features are generated by employing several non-linear transformations upon the base features of RNA and protein; finally, a feature selection based on random forest are employed to reduce the dimensions of the generated features. A series of experimental results on several widely used public datasets show that the prediction model using CFRP features is superior to the one using traditional k-mer features. It suggests that complex features generated by the CFRP framework are more descriptive than traditional k-mer features. The CFRP model is also compared with other state-of-the-art methods, and the results show that it could achieve better performance in terms of most of the tested metrics. In conclusion, the propose CFRP method could generate a set of complex features that is more informative that k-mer features. It would be conducive to build a prediction model of ncRNA-protein interaction with more powerful performance. The idea of constructing complex features might be extended to predicting other kinds of molecular interactions such as protein-protein interaction in the field of bioinformatics.

## Author Contributions

QD and MG designed the project. MG and XD developed the feature construction and selection methods. ZT and YF analyzed the result. QD and ZT conceived the experiment and wrote the manuscript. All authors read and approved the manuscript.

### Conflict of Interest Statement

The authors declare that the research was conducted in the absence of any commercial or financial relationships that could be construed as a potential conflict of interest.
